# The Germ Doctrine and Septicæmia

**Published:** 1877-11

**Authors:** 


### THE GERM DOCTRINE AND SEPTICÆMIA.

Dr. M. A. E. Wilkinson, President of the British Medical Association, in his address, spoke of the germ doctrine and its applications. He said:—

We will inquire how it stands with this doctrine in regard to traumatic septicæmia and pyæmia. You are all aware that foul, ill-conditioned wounds are attended with severe, often fatal, symptoms, consisting essentially of fever of a remittent type, tending to run on to the formation of embolic inflammations and secondary abscesses.

The notion that septicæmia is produced by bacteria, and the *rationale* of the antiseptic treatment which is based thereupon, is founded on the following series of considerations:—

1. It is known that decomposing animal substances, blood, muscle, and pus, develop, at an early stage of the process, a virulent poison, which, when injected into the body of an animal, produces symptoms similar to those of clinical septicæmia. This poison is evidently not itself an organism; it is soluble, or at least diffusible, in water, and it is capable, by appropriate means of being separated from the decomposing liquid and its contained organisms. When thus isolated, it behaves like any other chemical poison; its effects are proportionate to the dose, and it has not the least power of self-multiplication in the body. To this substance Dr. Burdon-Sanderson has given the appropiate name of pyrogen. It is the only known substance which produces a simple uncomplicated paroxysm of fever, beginning with a rigor, followed by a rise of temperature, and ending (if the dose be not too large) in defervesence and recovery.

2. We know further, from the evidence I have laid before you, that decomposition cannot take place without bacteria, and that bacteria are never produced spontaneously, but originate invariably from germs derived from the surroundiug media. We are warranted by analogy in regarding pyrogen as the product of a special fermentation taking place in decomposing albuminoid mixtures, but we cannot name the particular organism, nor the particular albuminoid compound which are mutually engaged in the process.

3. In the third place, we know that when a wound becomes unhealthy, as surgeons term it, the discharges become offensive, in other words, decomposed, and when examined under the microscope they are found to swarm with organisms resembling those found in all decomposing fluids. Meanwhile the patient becomes feverish, and suffers from the train of symptoms which we call septicæmia.

It is a natural inference that what takes place in decomposing blood or muscle in the laboratory, takes place also in the serous discharges and dead tissues of the wound. These become infected from the surrounding air, or from the water used in the dressings, with septic organisms; on that follows decomposition and the production of the septic poison, or pyrogen; the poison is absorbed into the blood, and septicæmia ensues.

It was the distinguished merit of Lister to perceive that these considerations pointed to a means of preventing septicæmia. He argued that, if you could prevent the access of septic organisms to the wound, or destory them there, you would prevent decomposition, prevent the production of the septic poison, and thus obviate the danger of septicæmia.

